# Long-term cellular immune response in immunocompromised unvaccinated COVID-19 patients undergoing monoclonal antibody treatment

**DOI:** 10.3389/fimmu.2022.980698

**Published:** 2022-10-13

**Authors:** Laura Thümmler, Margarethe Konik, Monika Lindemann, Neslinur Fisenkci, Michael Koldehoff, Anja Gäckler, Peter A. Horn, Fotis Theodoropoulos, Christian Taube, Markus Zettler, Olympia Evdoxia Anastasiou, Peer Braß, Sarah Jansen, Oliver Witzke, Hana Rohn, Adalbert Krawczyk

**Affiliations:** ^1^ Department of Infectious Diseases, West German Centre of Infectious Diseases, University Medicine Essen, University Hospital Essen, University Duisburg-Essen, Essen, Germany; ^2^ Institute for Transfusion Medicine, University Medicine Essen, University Hospital Essen, University Duisburg-Essen, Essen, Germany; ^3^ Department of Hematology and Stem Cell Transplantation, University Medicine Essen, University Hospital Essen, University of Duisburg-Essen, Essen, Germany; ^4^ Department of Hygiene and Environmental Medicine, University Medicine Essen, University Hospital Essen, University of Duisburg-Essen, Essen, Germany; ^5^ Department of Nephrology, University Medicine Essen, University Hospital Essen, University of Duisburg-Essen, Essen, Germany; ^6^ Department of Pneumology, University Medicine Essen-Ruhrlandklinik, University Duisburg- Essen, Essen, Germany; ^7^ Institute for Virology, University Medicine Essen, University Hospital Essen, University Duisburg-Essen, Essen, Germany

**Keywords:** COVID-19, immunosuppression, SARS-CoV-2, monoclonal antibody treatment, cellular immune response

## Abstract

Immunocompromised patients are at increased risk for a severe course of COVID-19. Treatment of severe acute respiratory syndrome coronavirus-2 (SARS-CoV-2) infection with anti-SARS-CoV-2 monoclonal antibodies (mAbs) has become widely accepted. However, the effects of mAb treatment on the long-term primary cellular response to SARS-CoV-2 are unknown. In the following study, we investigated the long-term cellular immune responses to SARS-CoV-2 Spike S1, Membrane (M) and Nucleocapsid (N) antigens using the ELISpot assay in unvaccinated, mAb-treated immunocompromised high-risk patients. Anti-SARS-CoV-2 mAb untreated though vaccinated COVID-19 immunocompromised patients, vaccinated SARS-CoV-2 immunocompromised patients without COVID-19 and vaccinated healthy control subjects served as control groups. The cellular immune response was determined at a median of 5 months after SARS-CoV-2 infection. Our data suggest that immunocompromised patients develop an endogenous long-term cellular immune response after COVID-19, although at low levels. A better understanding of the cellular immune response will help guide clinical decision making for these vulnerable patient cohorts.

## Introduction

The severe acute respiratory syndrome coronavirus 2 (SARS-CoV-2) has caused a pandemic of coronavirus disease 2019 (COVID-19), resulting in more than 530 million infected people and 6.3 million deaths (June 2022). Despite the availability of vaccines, the pandemic remains a global health burden (1). Many risk factors for the progression of COVID-19 to a severe and critical stage have been identified, including age, underlying comorbidities such as diabetes, obesity, chronic lung diseases, and immunodeficiency (2–4). Primary SARS-CoV-2 infections as well as breakthrough infections represent a potential risk for these vulnerable groups (5) resulting in a high burden of morbidity and mortality (6). During the early pandemic, COVID-19 patients were treated with SARS-CoV-2 convalescent plasma. Numerous studies were conducted, indicating that early onset of antiviral treatment is necessary to improve the course of disease and protect against a severe outcome (7–10). Later, monoclonal antibodies (mAb) against SARS-CoV-2 became available (11). Early treatment of SARS-CoV-2 infections with mAbs such as bamlanivimab (12) or a combination of the monoclonal antibodies casirivimab and imdevimab (11) has been shown to markedly reduce the risk of hospitalization or death among high-risk patients with COVID-19 (11, 12). However, the occurrence of novel SARS-CoV-2 variants of concern (VOCs) such as Alpha (Pango nomenclature B.1.1.7), Beta (B.1.351), Gamma (P1, B.1.1.28), Delta (B.1.617.2) and Omicron (B.1.1.529) led to an increase in the frequency of reinfection and vaccination breakthrough SARS-CoV-2 infections (3, 13, 14). Some of the mutations within the SARS-CoV-2 spike antigen are associated with immune escape, and thus a reduced effectivity of monoclonal antibodies against SARS-CoV-2 spike protein variants (15, 16). However, a recent study suggests that monoclonal antibody treatment, with respect to available antibody formulations and circulating viral variants, may provide favorable outcomes for mild to moderate COVID-19 in vulnerable patients, such as solid organ recipients (17).

Although the role of antibodies induced by immunization or additionally administrated early upon infection in those patients was already described, less is known about the cellular immune response in immunocompromised patients with primary or breakthrough infections and antibody treatment (18).

In the present study, we investigated the long-term cellular immune response in severely immunocompromised unvaccinated patients suffering from a SARS-CoV-2 infection and treated with the mAb bamlanivimab or a combination of the mAbs casirivimab and imdevimab in the early phase of infection. We compared the cellular immune response of these patients with those of vaccinated immunocompromised patients with a SARS-CoV-2 infection but without antibody treatment as well as vaccinated immunocompromised patients and immunocompetent volunteers without SARS-CoV-2 infection.

## Methods

### Study subjects and sampling

In the present study, we investigate the long-term cellular immune response against SARS-CoV-2 spike (S), Membrane (M) and Nucleocapsid (N) antigens in immunocompromised patients with primary SARS-CoV-2 infection after early mAb treatment (group 1) up to 5 months after COVID-19. Vaccinated immunocompromised patients with COVID-19 (group 2), as well as vaccinated immunocompromised patients (group 3) and vaccinated healthy volunteers (group 4) without COVID-19 served as controls. All immunocompromised patients (group 1-3) had a medical condition associated with secondary severe immunodeficiency. Patients suffering from a primary SARS-CoV-2 infection (group 1) were treated early with monoclonal antibodies (mAbs) bamlanivimab (LY-CoV555, Eli Lilly) or casirivimab/imdevimab (Ronapreve, Roche and Regeneron), which both bind to the SARS-CoV-2 spike protein. Group 1 included 12 non-vaccinated patients. Of the 12 patients, 8 were treated with 700 mg bamlanivimab (concentration 35 mg/ml) and 4 with a combination of 1200 mg casirivimab/imdevimab (concentration of 120 mg/ml each). Antibodies were administrated intravenously. The group consisted of 2 men and 10 women, and the median age was 57 years (range 31-78). The cellular immune response in the first group was examined at a median of 146 days (range 74-182) after mAb therapy. One of the patients had breast cancer, three had a kidney transplantation (median since transplantation 5.6 years, range 4 months – 10 years), seven had a lung transplantation (median since transplantation 10.2 months, range 4 - 25 months), one prostate cancer and one cachexia. In this first group, the three kidney transplant patients had concomitant arterial hypertension and an impaired renal function. In the lung transplant recipient group, one patient had coronary artery disease as an additional risk factor for a severe COVID-19 course. Except for one patient, all solid organ transplant patients had triple immunosuppressive therapy containing prednisone, the calcineurin inhibitor (CNI) tacrolimus, and mycophenolic acid (MMF) or the mTOR inhibitor everolimus. One renal transplant patient had triple immunosuppressive therapy containing prednisolone, MMF, and belatacept.

Group 2 included 10 immunocompromised, vaccinated patients with a SARS-CoV-2 infection. All patients were vaccinated with an mRNA vaccine (BioNTech or Moderna). The group was composed of seven men and three women and the median age was 59 years (range 20-69) after hematopoietic stem cell transplantation (HSCT). HSCT took place at a median of 2.9 years (range 0.9-17) prior to blood collection and all patients achieve complete remission of their underlying hematologic malignancy. Three patients had coronary artery disease as additional COVID-19 risk factor, one patient had grade I obesity, and one had a history of chronic obstructive pulmonary disease and splenectomy. 7 patients had developed graft versus host disease (GVHD) after HSCT and were treated immunosuppressive with steroids with or without CNI for this purpose. One patient received an mTor inhibitor plus steroids. The cellular immune response was explored at a median of 145 days (range 61-230) after infection.

Group 3 included 14 immunocompromised, vaccinated patients without SARS-CoV-2 infection. All patients were vaccinated with an mRNA vaccine (n=13 Comirnaty, BioNTech/Pfizer; n=1 Spikevax, Moderna). The group contained six men and eight women with a median age of 55 years (range 21-64). Of the 14 patients, four had a HSCT a median of 4.3 years (range 1.3-16.1) prior to testing and ten had a kidney transplant at a median of 3.1 years (range 0.09-10.5) prior to blood collection. The cellular immune response in this group was examined at a median of 87 days (range 16-238) after vaccination. 3 HSCT patients were on dual immunosuppressive therapy (steroid, with CNI or MMF) because of persistent GVHD; one patient had additional arterial hypertension and diabetes mellitus as risk factors for severe COVID-19 progression. All except one kidney transplant recipients had concomitant arterial hypertension and impaired renal function and received triple immunosuppressive therapy with prednisolone, MMF, and CNI. One patient received triple immunosuppressive therapy with prednisolone, MMF, and belatacept.

Group 4 included 14 healthy volunteers after the third SARS-CoV-2 vaccination with an mRNA SARS-CoV-2 vaccine (n=3 Comirnaty, BioNTech/Pfizer; n=11 Spikevax, Moderna). The group was composed of six men and eight women and the median age was 50 years (range 29-65). They were tested at a median of 47 days (range 30-72) after the third vaccination.

There were no significant differences between the different cohorts with respect to the known COVID-19 related risk factors being sex, age, and lymphocyte count (**Table 1**).

**Table 1:** Overview of the study cohort.

group	1	2	3	4	p-value
**Antibody therapy?** **(Y: yes, N: no)**	Y	N	N	N	
**Infected?** **(Y: yes, N: no)**	Y	Y	N	N	
**Vaccinated?** **(Y: yes, N: no)**	N	Y	Y	Y	
**Severely Immunocompromised?** **(Y: yes, N: no)**	Y	Y	Y	N	
**Total number**	12	10	14	14	
**number of women**	10	3	8	8	*p* = 0.09
**number of men**	2	7	6	6	
**median age [years] (range)**	57 (31-78)	59 (20-69)	55 (21-64)	50 (29-65)	*p* = 0.23
**Count of lymphocytes [x103/µl]** **Mean (SD)**	8.8 (6.4)	9.6 (13.0)	14.1 (5.8)	12.1 (5.0)	*p* = 0.29
**Interval infection/vaccination– blood collection** **Mean (range)**	146 days (74-182)	145 days (61-230)	87 days (16-238)	47 days (30-72)	*p< 0.001*
**SARS-CoV-2 vairant or SARS-CoV-2 vaccine**	SARS-CoV- 2 D614G (wild type)	SARS-CoV- 2 D614G (wild type) 8x Comirnaty^®^ (BioNTech/Pfizer) 2x Spikevax^®^ (Moderna)	13x Comirnaty^®^ (BioNTech/Pfizer) 1x Spikevax^®^ (Moderna)	3x Comirnaty^®^ (BioNTech/Pfizer) 11x Spikevax^®^ (Moderna)	
**Number of SARS-CoV-2 vaccinations**	0	2	2	3	

Characteristics of the four different study groups. Groups 1-3 include the different cohorts of patients in terms of monoclonal antibody treatment, SARS-CoV-2 infection, and SARS-CoV-2 vaccination; group 4 represents the healthy control group (SARS-CoV-2 vaccination, without SARS-CoV-2 infection). Comparison between COVID-19 related risk factors sex, age and lymphocyte counts was performed using the Kruskal Wallis test. Statistical significance was set at the level of p < 0.05.

The study was conducted according to the Helsinki principles and was approved by the local ethics committee of the University Hospital Essen, Germany (20-9225-BO, 20-9254-BO, and 20-9374-BO). All subjects provided informed consent to participate in the study.

### T cell ELISpot assays for S1-, M-, and N-derived SARS-CoV-2 peptides

To analyze SARS-CoV-2 specific cellular immune responses, we performed interferon gamma (IFN-γ) enzyme-linked immunospot (ELISpot) assays as previously described (19). 250,000 peripheral blood mononuclear cells (PBMC) were cultured in the presence or absence of the PepTivator^®^ SARS-CoV-2 membrane (M) protein, nucleocapsid (N) protein (600 pmol/mL of each peptide, Miltenyi Biotec, Bergisch Gladbach, Germany) or the S1 protein (4 µg/ml, Sino Biological, Wayne, PA, USA) (each in single cell culture) in 150 µL of AIM-V^®^. The peptide mixes corresponding to the M and N proteins cover the complete sequence of the glycoproteins. The S1 protein is a recombinant protein expressed in HEK293 cells and covers the sequence of aa 1 to aa 692 of the spike protein subunit 1. The peptide pools consisted of 15-mer sequences with an overlap of 11 amino acids. After 19 h of incubation at 37°C, IFN-γ production was measured as previously described (19). The spot numbers were evaluated by an ELISpot reader (AID Fluorospot; Autoimmun Diagnostika GmbH, Strassberg, Germany). From duplicate cell cultures, the mean value was considered. SARS-CoV-2 specific spots were determined as stimulated minus non-stimulated values (spot increment). The negative controls had an average of 0.21 spots (range 0-3) and their three-fold standard deviation was 3 x 0.67 spots = 2.01 spots (which we considered as background for the negative controls). As we used increment values, a three-fold higher value versus background means 3 x 2.01 spots minus 1 x 2.01 spots, which is 4.02 spots increment. We therefore chose a cut-off point of 4 as positivity.

### Statistical analysis

Statistical analysis was performed using GraphPad Prism 9.3.1 software (San Diego, CA, USA). We used the Kruskal-Wallis-test and the Mann-Whitney U test for numerical variables. Fisher’s exact test was used for categorical variables. Two-sided *p* values < 0.05 were considered significant.

## Results

In the present study, we examined the long-term cellular immune response unvaccinated severely immunocompromised patients suffering from a SARS-CoV-2 infection after treatment with bamlanivimab or a combination of the monoclonal antibodies casirivimab and imdevimab early after infection using ELISpot assay. We also investigated the cellular immune response in vaccinated immunocompromised patients after SARS-CoV-2 infection as well as in vaccinated immunocompromised patients and immunocompetent volunteers without a history of SARS-CoV-2 infection.

**Figure 1:**
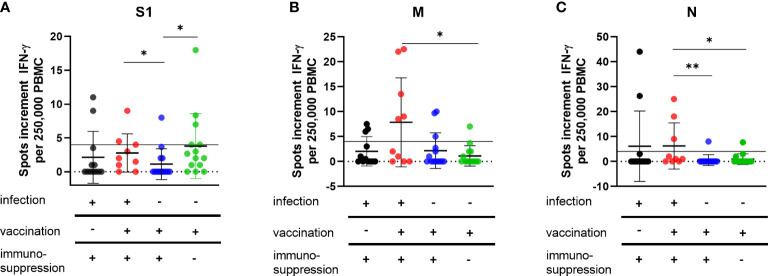
Cellular immunity to SARS-CoV-2 antigens in vaccinated and/or recovered immunosuppressed individuals. Cellular immune responses towards SARS-CoV-2 S1 **(A)**, M **(B)** and N **(C)** antigens were determined by IFN-γ ELISpot assay. Please note that the scales differ. Horizontal lines indicate mean values, error bars indicate the standard deviation (SD). The dotted gray line represents the assay cut-off. Differences between the groups were compared by Mann-Whitney U test (*p < 0.05).

Unvaccinated immunocompromised patients with SARS-CoV-2 infection and early mAb treatment (group 1) showed a similar cellular immune response to all stimuli in the ELISpot assay to vaccinated immunocompromised patients with a SARS-CoV-2 infection (group 2) (**Figures 1A–C**). The measured mean values of spots increment after stimulation with spike S1 protein were 2.1 in group 1 and 2.8 in group 2 (*p* = 0.2) (**Figure 1A**), after stimulation with M protein 2.0 in group 1 and 7.9 in group 2 (*p* = 0.09) (**Figure 1B**) and after stimulation with N protein 6.1 in group 1 and 6.2 in group 2 (*p* = 0.2) (**Figure 1C**). In particular, the cellular immune response in group 1 and 2 was higher than in vaccinated immunocompromised patients (group 3) (**Figures 1A–C**). Significant differences in spots increment were observed between group 2 and 3 after stimulation with S1 protein (2.8 in group 2 and 1.1 in group 3 (*p* = 0.04)) (**Figure 1A**) and N protein (6.2 in group 2 and 0.6 in group 3 (*p* = 0.002)) (**Figure 1C**). As expected, healthy immunocompetent vaccinated volunteers (group 4) showed a higher cellular immune response than vaccinated immunocompromised patients (group 3) (**Figure 1A**). The spots increment after stimulation with the spike S1 protein were 1.1 in group 3 and 3.8 in group 4 (*p* = 0.01) (**Figure 1A**). No significant differences between groups 3 and 4 could be observed after stimulation with M protein (3.7 in group 3 and 1.1 in group 4 (*p* = 0.5) or N protein (0.6 in group 3 and 0.9 in group 4 (*p* = 0.6) (**Figures 1B, C**).

Interestingly, after mAb treatment (group 1), the frequency of single and combined positive cellular response to S1, M, or N proteins was lower than in the vaccinated cohort with SARS-CoV-2 infection (group2) (S1: 14% vs. 30%; M: 21% vs. 50%; N: 14% vs. 30%, combined: 33% vs. 60% statistically not significant as calculated with the Fischer´s exact test). In the groups vaccinated against SARS-CoV-2 without COVID-19, only one of 14 immunocompromised patients (group 3) developed a positive response to S1 (7%), compared with 5 of 14 (35%) in the volunteer group (group 4). Immune responses against the M antigen could be detected in three volunteers from group 3 and two from group 4 and against the N antigen in one volunteer from group 3 and 4, respectively. All of these volunteers had no documented SARS-CoV-2 infection.

## Discussion

In this paper, we present the profiling of cellular immunity in a cohort of immunocompromised, unvaccinated patients who developed COVID-19 and thus were treated with bamlanivimab or with the combination therapy casirivimab and indevimab. A cohort of immunocompromised patients vaccinated against SARS-CoV-2 who developed COVID-19, as well as immunized immunocompromised patients or healthy participants without COVID-19, served as controls. Our data suggest that immunocompromised patients may develop an endogenous long-term cellular immune response after COVID-19. The observed T cell-mediated immunity against the spike protein in unvaccinated immunocompromised patients after mAb therapy, seems to be blunted compared to vaccinated and mAb untreated immunocompromised patients with COVID-19. Consistent with this finding, cellular immune responses in our patient cohort were lower after mAb treatment compared with previously published results from immunosuppressed cohorts after COVID-19 but without mAb treatment (20–24).

Early treatment of SARS-CoV-2 infection in high-risk cohorts with mAbs is widely accepted, and mAbs clinical trials have reported overall reduced hospitalization rates in patients with mild to moderate COVID-19 (25–29). While many studies focused on the clinical efficacy of the treatment, its effects on long-term immunologic responses to the virus are largely unknown (12, 17, 30–33). The optimal use of these therapeutic options requires a sophisticated understanding of their effects on both the virus and the host immune system. For a long time, anti-SARS-CoV-2 T cell immunity was considered less important than specific antibodies as a parameter for immune protection in patients at risk of severe COVID-19 (34). However, the humoral anti-SARS-CoV-2 response declines over time, whereas SARS-CoV-2-specific T cell immunity appears to persist longer, even in seronegative convalescents (35–38). To our knowledge, no study has explored the effect of mAb treatment on cellular immunity in severely immunocompromised patients at risk for severe COVID-19 and only two studies explored the effect of treatment on humoral immunity (39, 40). Both studies demonstrated that passive immunization of COVID-19 patients with anti-S monoclonal IgG preparations profoundly suppressed the induction of the endogenous anti-S IgM response and, to a lesser extent, the anti-N IgG response. It is noteworthy that not only immunosuppressed patients were included in those analyses, as these patients would likely exhibit a reduced immune response due to immunosuppression (41–45). Anti-SARS-CoV-2 mAb preparations are reportedly able to reduce viral load (46, 47). Reduction of viral load in the early stage of infection might be expected to result in reduced immune responses. Additionally, stronger SARS-CoV-2 specific T cell responses are well documented in patients who had recovered from more severe symptoms of COVID-19 (48–51). Therefore, it seems possible that after mAbs treatment, the improvement in COVID-19 course is causative of the decreased immunologic response.

## Conclusion

Most of the immunosuppressed, non-vaccinated COVID-19 patients treated with monoclonal antibodies within the present study developed no SARS-CoV-2 specific T-cell response. The adaptive immune response is an important factor in the clinical course after SARS-CoV-2 infection and may protect from reinfections. Deeper immunophenotyping of immunocompromised patients after mAb therapy will be important in expanding knowledge about long-term immunity to SARS-CoV-2. Its understanding is not only essential to evaluate the potential effect of COVID-19 treatment on future reinfection but also crucial for further risk assessment especially in the high-risk cohort of immunocompromised patients.

## Data availability statement

The raw data supporting the conclusions of this article will be made available by the authors, without undue reservation.

## Ethics statement

The study was conducted according to the Helsinki principles and was approved by the local ethics committee of the University Hospital Essen, Germany (20-9225-BO, 20-9254-BO, and 20-9374-BO). All subjects provided informed consent to participate in the study.

## Author contributions

Conceptualization, MaK, ML, HR, OW, PAH, and AK; methodology, LT, MaK, AG, FT, and CT; validation, AK, HR, and OW; investigation, LT, NF, PB, and SJ; resources, MaK, MiK, AG, CT, and MZ; data curation, LT, MZ, OA, and OW; writing—original draft preparation, LT, HR, and AK; writing—review and editing, LT, HR, ML, and AK; visualization, LT, HR, and AK; supervision, HR and AK; funding acquisition, OW, HR, and AK. All authors have read and agreed to the published version of the manuscript.

## Funding

OW received funding from the Rudolf Ackermann-Stiftung, AK and HR received funding from the Stiftung Universitätsmedizin Essen. The publication is support by the Open Access Publication Fund of the University of Duisburg-Essen.

## Acknowledgments

We feel deep gratitude to the patients who donated their blood samples and clinical data for this project.

## Conflict of interest

The authors declare that the research was conducted in the absence of any commercial or financial relationships that could be construed as a potential conflict of interest.

## Publisher’s note

All claims expressed in this article are solely those of the authors and do not necessarily represent those of their affiliated organizations, or those of the publisher, the editors and the reviewers. Any product that may be evaluated in this article, or claim that may be made by its manufacturer, is not guaranteed or endorsed by the publisher.
